# Two cases of circulatory collapse due to suspected remimazolam anaphylaxis

**DOI:** 10.1186/s40981-022-00508-5

**Published:** 2022-03-06

**Authors:** Satoshi Uchida, Daiki Takekawa, Masato Kitayama, Kazuyoshi Hirota

**Affiliations:** Department of Anesthesiology, Hirosaki University Graduate School of Medicine, 5 Zaifu-cho, Hirosaki, 036-8562 Japan

**Keywords:** Remimazolam, Anaphylaxis, Tryptase

## Abstract

**Background:**

Remimazolam was approved in Japan in January 2020. We report two cases of circulatory collapse due to suspected remimazolam anaphylaxis during anesthetic induction.

**Case presentation:**

Case 1: A 74-year-old male was scheduled for debridement and skin grafting for a severe burn injury. We induced anesthesia with 4 mg of remimazolam and 20 mg of ketamine. The patient subsequently developed treatment-resistant severe hypotension.

Case 2: A 59-year-old male was scheduled for laparoscopic-assisted sigmoid colectomy. We induced anesthesia with 9 mg of remimazolam. Within a few minutes, the patient developed treatment-resistant severe hypotension.

As serum tryptase was elevated in both cases and only intravenous administration of adrenaline was effective, we considered the circulatory collapse might be due to anaphylaxis.

**Conclusion:**

We experienced two cases of circulatory collapse due to suspected remimazolam anaphylaxis during anesthetic induction. The prevalence of remimazolam anaphylaxis is not yet known, and further research is needed.

## Background

Remimazolam besylate (Anerem®), a novel benzodiazepine characterized by its ultra-short acting property with flumazenil as a specific antagonist, was approved in Japan in January 2020 [1]. Although the safety of remimazolam has been evaluated [2, 3], the frequency of related anaphylaxis is not yet known and one published report describes a case of remimazolam anaphylaxis [4]. Here, we report two cases of circulatory collapse due to suspected remimazolam anaphylaxis during anesthetic induction with elevated serum tryptase.

## Case presentation

### Case 1

A 74-year-old male (height, 157 cm; weight, 78 kg) with no history of drug allergy was scheduled for a third debridement and skin grafting for a severe burn injury. He had a past medical history of hypertension and diabetes. The first debridement was performed one month before, and anesthesia was induced and maintained with remimazolam, propofol, ketamine, fentanyl, and rocuronium without any problems during the first surgery. The second debridement with tracheostomy was performed 9 days before, but the operation was discontinued because non-sustained ventricular tachycardia occurred due to the development of intraoperative hypothermia. Anesthesia was induced and maintained with propofol, ketamine, fentanyl, and rocuronium without any problems during the first surgery.

Before anesthetic induction for the third surgery, the patient’s vital signs were as follows: blood pressure, 162/64 mmHg; heart rate, 81 bpm; and SpO_2_, 94%. We connected a ventilator circuit to the tracheostomy tube without any problem. We induced anesthesia with 4 mg of remimazolam and 20 mg of ketamine. His systolic blood pressure quickly dropped to 30–40 mmHg, and SpO_2_ dropped to 73% without any S–T change in electrocardiography. Wheezing and any other abnormal respiratory sounds were not audible.

**Fig. 1:**
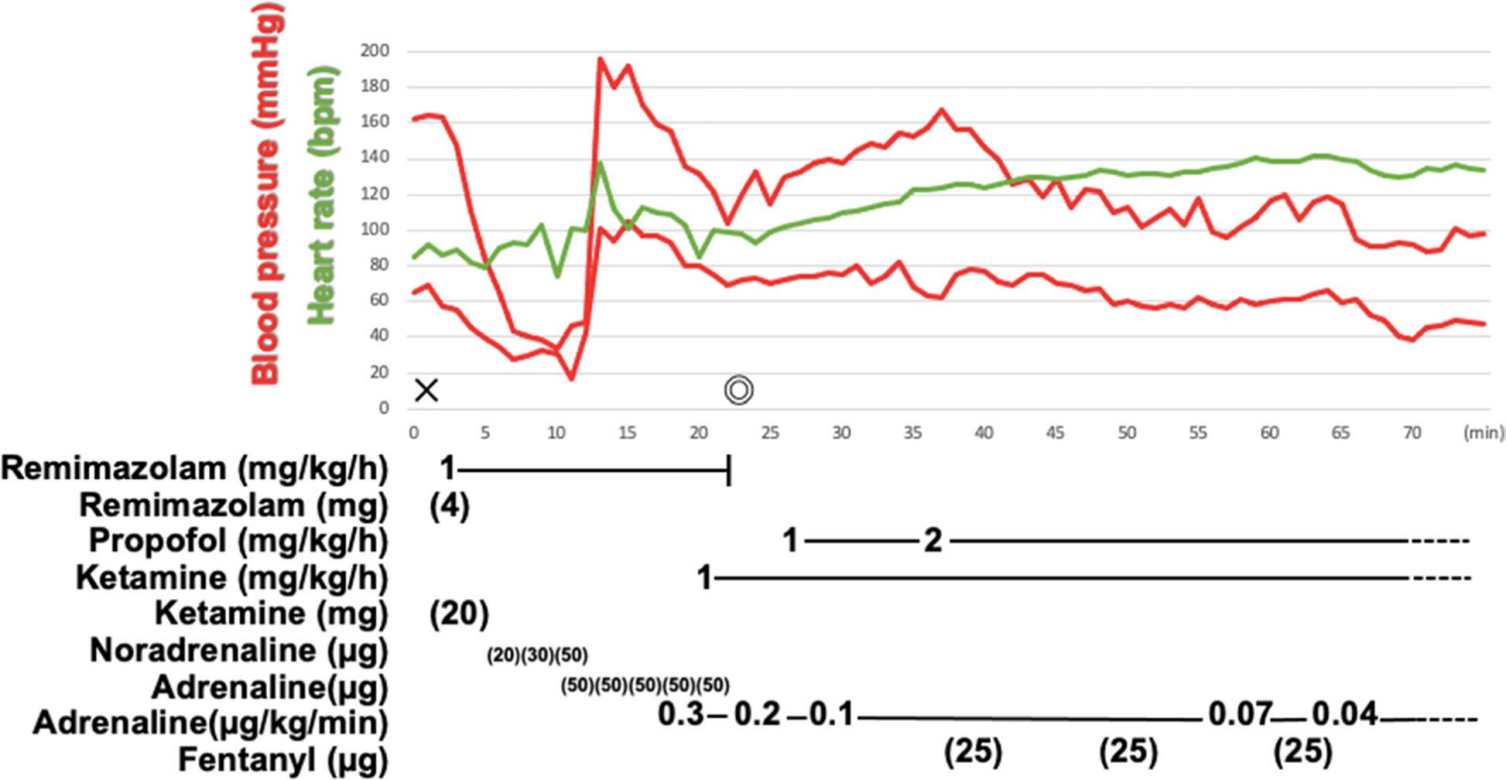
Time course of general anesthesia in case 1. We stopped adrenaline infusion at the end of anesthesia

We administrated 100 μg of noradrenaline in multiple divided doses, but hemodynamics did not change drastically. We then administered 50 μg of intravenous adrenaline repeatedly (total 250 μg) and 2000 mL of crystalloid, and his blood pressure and SpO_2_ returned to 115/70 mmHg and 98%. At this point, we could not diagnose anaphylaxis because skin symptoms could not be confirmed due to the burn injury and there were no respiratory symptoms. We suspected that this severe hypotension was caused by hypovolemic and/or septic shock due to burn wound infection, which was emphasized by administration of anesthetics. Considering that the previous surgery had been canceled halfway, we decided to continue the surgery. Anesthesia was maintained with propofol, ketamine, and fentanyl with 0.03–0.2 μg/kg/min of continuous intravenous infusion of adrenaline during the surgery (Fig. 1).

The surgery was completed, and a subsequent blood test suggested that circulatory collapse after anesthetic induction was due to anaphylaxis. Serum tryptase level was elevated from baseline sample (2.9 μg/L) to acute sample (8.3 μg/L).

### Case 2

A 59-year-old male (height, 176 cm; body weight, 52 kg) with no history of drug allergy was scheduled for a laparoscopic-assisted sigmoid colectomy. He had a past medical history of diabetes. He had undergone esophageal bypass surgery for cervical esophageal cancer and open liver biopsy. Anesthesia was induced and maintained with propofol, remifentanil, ketamine, and rocuronium during the previous surgery.

Before anesthetic induction, his vital signs were as follows: blood pressure, 124/84 mmHg; heart rate, 88 bpm; and SpO_2_, 99%. We induced anesthesia with 9 mg of remimazolam divided into three doses. The patient then complained of discomfort. Within a few minutes, he developed sinus tachycardia of 105 bpm, and we were unable to measure blood pressure with the manchette method due to hypotension and body movement. We were able to palpate the radial artery pulse slightly. Although the patient seemed to lose consciousness, the bispectral index was more than 90 and body movement continued. We administered 8 mg of ephedrine and intubated immediately after administration of 60 mg of propofol and 40 mg of suxamethonium. We catheterized the radial artery at the same time. Since the patient’s systolic blood pressure remained at 30–40 mmHg, ephedrine and phenylephrine were administered repeatedly, but hemodynamics did not change. Thus, we administered intravenous 50 μg of adrenaline repeatedly (total 300 μg), following which his blood pressure returned to 85/45 mmHg and hemodynamic was stabilized. After confirming hemodynamic stability, we started 2 mg/kg/h of continuous intravenous infusion of propofol for sedation.

**Fig. 2:**
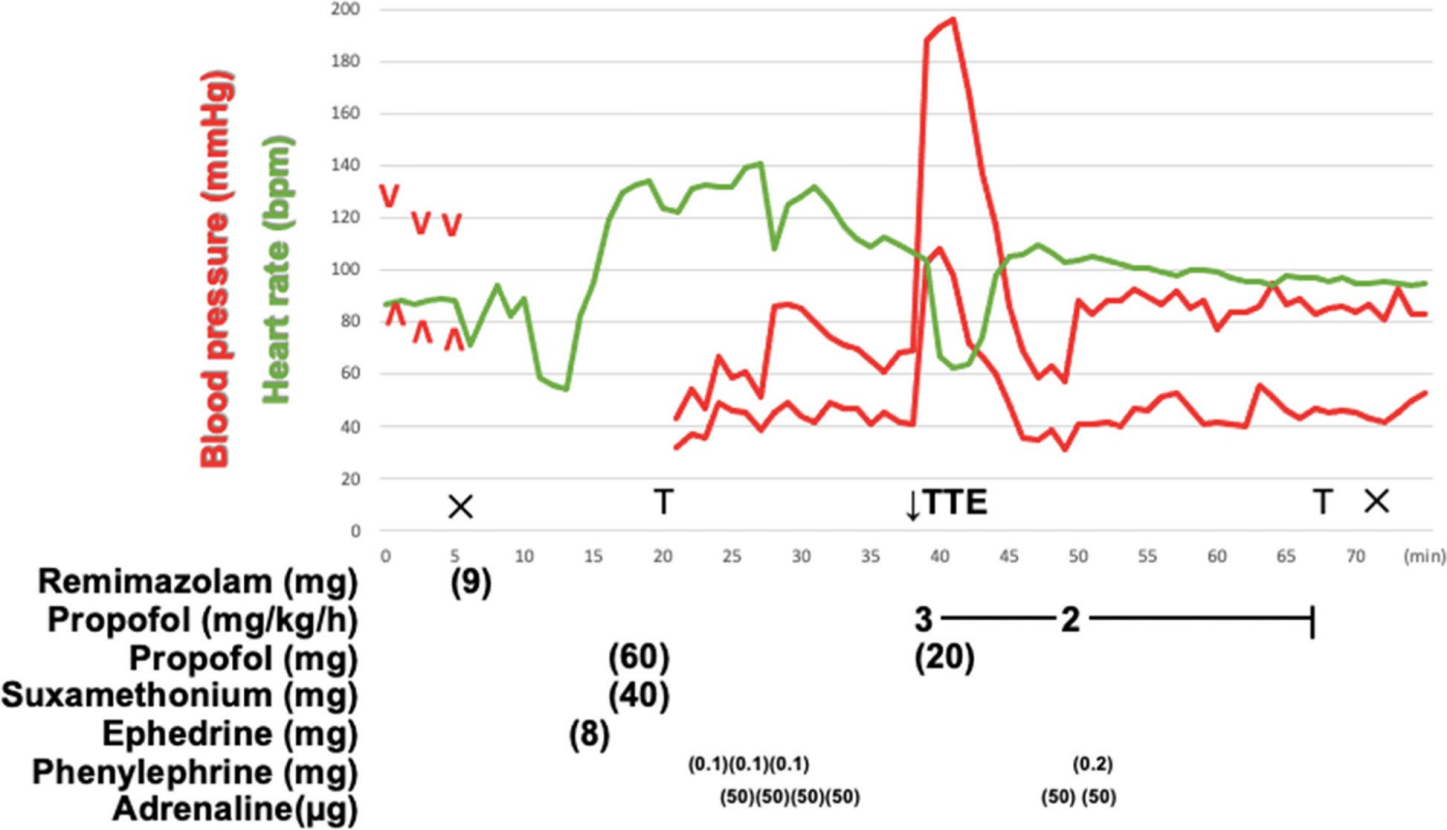
Time course of general anesthesia in case 2. We were unable to measure blood pressure with the manchette method after remimazolam infusion. It was due to hypotension and the body movement. Following then, we catheterized the radial artery, but it took some time. We were able to palpate the radial artery pulse slightly during this period. TTE: Transthoracic echocardiography

At this point, we could not diagnose anaphylaxis because skin and respiratory symptoms could not be confirmed. We performed echocardiography, but no segmental asynergy, right ventricular dilatation, or inferior vena cava collapse was observed. Troponin T was not elevated (0.001 ng/mL). The operation was discontinued, and subsequent blood test suggested anaphylaxis. Serum tryptase was elevated from the baseline sample (4.1 μg/L) to the acute sample (7.8 μg/L). The time course of general anesthesia was shown in Fig. 2. We suspected remimazolam anaphylaxis considering the onset situation. However, the skin prick and intradermal tests were negative for remimazolam.

One month later, the operation was rescheduled, and anesthesia was induced and maintained with propofol, ketamine, fentanyl, and rocuronium. This operation was completed without any problems.

## Discussion

We present two cases of circulatory collapse due to suspected remimazolam anaphylaxis. Since serum tryptase was elevated in both cases, we diagnosed the circulatory collapse as due to anaphylaxis. In case 1, we induced anesthesia with 4 mg of remimazolam and 20 mg of ketamine, and then circulatory collapse occurred. Anaphylaxis due to ketamine is extremely rare [5]. Additionally, we maintained anesthesia with ketamine during surgery, while titrating the dose of adrenaline. Thus, it was unlikely that ketamine induced anaphylaxis, and remimazolam was more likely the cause. As remimazolam was used in the first debridement, the patient may have become sensitized to it. However, as we did not conduct any allergy tests due to lack of patient’s consent, we could not confirm this diagnosis.

In case 2, we induced anesthesia with only 9 mg of remimazolam, after which circulatory collapse occurred. However, skin prick and intradermal tests were negative for remimazolam. A systematic review and meta-analysis reported that the sensitivity and specificity for the skin-prick test were 85 and 77%, respectively, while the sensitivity and specificity for the intradermal test were 60–79% and 68%, respectively [6]. Thus, the possibility of remimazolam anaphylaxis was not completely ruled out. Considering the situation at the onset of anaphylaxis, remimazolam was likely the causative drug.

Anaphylaxis is often diagnosed through clinical criteria. During general anesthesia, it is more difficult to recognize the symptoms of anaphylaxis, such as hypotension and respiratory symptoms, because anesthetics and tracheal intubation mask these symptoms and patients are unable to complain of them. Additionally, the skin symptom may not always appear in the patient with anaphylaxis under general anesthesia [7]. Therefore, obtaining paired acute-to-baseline serum tryptase level is recommended [7, 8]. Serum tryptase levels of acute phase > ([1.2×baseline tryptase] + 2) μg/L was reported to be a clinically significant rise, and the sensitivity and specificity of this value were 75 and 86%, respectively [9]. As our two cases met this criterion, we suspected that circulatory collapse was due to anaphylaxis. However, we should have suspected anaphylaxis during the surgeries, because only intravenous administration of adrenaline was effective to both patients. In case 1, if we could have suspected anaphylaxis, we could also discontinue ketamine as a suspected drug.

Remimazolam anaphylaxis due to cross-reactivity with midazolam has been reported [4]. The only structural difference between midazolam and remimazolam is that the latter has an ester bond in the side chain. In case 2, however, there was no history of midazolam use in addition to remimazolam. Therefore, we speculate that remimazolam anaphylaxis may occur not only for structural reasons, but also for other factors. Anelem®, as well as ByFavo™ and Aptymida™, remimazolam used in the USA and UK, respectively, contain dextran 40 as an additive. The prevalence of dextran 40 anaphylaxis is reported to be 0.003%, and even a small dose of dextran solution could induce anaphylaxis [10]. Thus, it is possible that the anaphylaxis was due to dextran 40.

Dextran anaphylaxis is known to be caused by an anti-dextran antibody that is present in most human bodies [11]. The anti-dextran antibody is induced by dextran contaminants in sugar, dextran in dental plaque, the cell wall of intestinal bacteria, pneumococci, etc., which means that we are sensitized to dextran in daily life [12]. On the other hand, skin test is reported not to be useful for dextran allergy [13]. Thus, the causative drug for anaphylaxis may have been dextran 40 added to remimazolam, especially in case 2.

## Conclusion

We experienced two cases of circulatory collapse due to suspected remimazolam anaphylaxis during anesthetic induction. As dextran 40 is added to remimazolam, clinicians should be careful for patients with allergy to dextran or products containing dextran when using remimazolam. Additionally, the prevalence of remimazolam anaphylaxis is not yet known and further research is needed.

## Data Availability

Not applicable.
